# Tracking Multiple Video Targets with an Improved GM-PHD Tracker

**DOI:** 10.3390/s151229794

**Published:** 2015-12-03

**Authors:** Xiaolong Zhou, Hui Yu, Honghai Liu, Youfu Li

**Affiliations:** 1 College of Computer Science and Technology, Zhejiang University of Technology, Hangzhou 310023, China; zxl@zjut.edu.cn; 2 School of Computing, University of Portsmouth, Portsmouth PO1 3HE, UK; 3 School of Creative Technologies, University of Portsmouth, Portsmouth PO1 2DJ, UK; hui.yu@port.ac.uk; 4 Department of Mechanical and Biomedical Engineering, City University of Hong Kong, Hong Kong, China; meyfli@cityu.edu.hk

**Keywords:** robot vision, video targets tracking, probability hypothesis density, weight penalization, multi-feature fusion

## Abstract

Tracking multiple moving targets from a video plays an important role in many vision-based robotic applications. In this paper, we propose an improved Gaussian mixture probability hypothesis density (GM-PHD) tracker with weight penalization to effectively and accurately track multiple moving targets from a video. First, an entropy-based birth intensity estimation method is incorporated to eliminate the false positives caused by noisy video data. Then, a weight-penalized method with multi-feature fusion is proposed to accurately track the targets in close movement. For targets without occlusion, a weight matrix that contains all updated weights between the predicted target states and the measurements is constructed, and a simple, but effective method based on total weight and predicted target state is proposed to search the ambiguous weights in the weight matrix. The ambiguous weights are then penalized according to the fused target features that include spatial-colour appearance, histogram of oriented gradient and target area and further re-normalized to form a new weight matrix. With this new weight matrix, the tracker can correctly track the targets in close movement without occlusion. For targets with occlusion, a robust game-theoretical method is used. Finally, the experiments conducted on various video scenarios validate the effectiveness of the proposed penalization method and show the superior performance of our tracker over the state of the art.

## 1. Introduction

Tracking targets in video is an ever-increasing field of research with a wide spectrum of applications in vision-based robotic intelligence, including robot navigation, intelligent surveillance, human behaviour understanding, human-robot interactions, and so on. Despite many excellent research works [1,2,3,4,5] having been explored, an effective and accurate solution to the problem remains challenging.

Recently, the random finite set approach for target tracking [6,7,8,9,10,11,12,13,14,15,16,17] has attracted considerable attention. The probability hypothesis density (PHD) filter [6] uses the first-order statistical moment of the multi-target posterior density, providing a computationally-tractable alternative to data association. However, it is generally intractable due to the “curse of dimensionality” in numerical integration. The Gaussian mixture PHD filter (GM-PHD) [7] does not suffer from this problem, because its posterior intensity function can be propagated analytically in time.

Although the GM-PHD filter originates from radar tracking [7,8,9,10], recently, it has been widely explored for visual tracking [11,12,13,14,15,16,17]. For simplicity, the GM-PHD filter-based tracker is called the GM-PHD tracker in this paper. For example, Pham *et al.* [11] used the GM-PHD tracker to track multiple objects from colour images. They showed that the PHD was proportional to the approximated density from colour likelihood. They also used this GM-PHD tracker to track 3D locations of heads of people using multiple cameras [12]. Wu and Hu [13] combined the modified detection method with the PHD filter to build a multi-target visual tracking framework. They first generated observations by detecting the foreground objects and then estimated the target state using a GM-PHD filter. Furthermore, Wu *et al.* [14] proposed an auction algorithm to calculate target trajectories automatically. Zhou *et al.* [15] incorporated entropy distribution into the GM-PHD filter to automatically and efficiently estimate the birth intensity and, finally, robustly tracked the newborn video targets. Furthermore, they used game theory to handle the mutual occlusion problem and proposed an integrated system to robustly track the multiple video targets [16]. Pollard *et al.* [17] used a homographic transformation to compensate the camera motion and to combine geometric and intensity-based criteria for object detection and combined the GMC-PHD filter to track the targets from an aerial video.

Despite significant progress of the GM-PHD tracker, robust and reliable tracking of multiple targets in video is still far from being solved, especially in noisy video data and tracking targets in close movement.

To eliminate the noisy data in the video, the tracker should have the ability of accurately determining the birth intensity of the newborn targets in the GM-PHD filter. Conventionally, the birth intensity must cover the whole state space [18] when no prior localization information on the newborn targets was available. Such a requirement entails a high computational cost and can easily be interfered by clutters. To remedy this, Maggio *et al.* [19] assumed that the birth of a target occurred in a limited space around the measurements. They drew the newborn particles from the centre of the measurement set. However, the proposed method could easily be interfered by clutters and the measurements originating from the survival targets. Recently, Zhou *et al.* [15] proposed an effective method based on entropy distribution to automatically and correctly estimate the birth intensity. They first initialized the birth intensity using the previously-obtained target states and measurements and then updated it using the currently-obtained measurements. The entropy distribution was incorporated to remove those noises that were irrelevant to the measurements, and the coverage rate was computed to further eliminate the noises.

**Figure 1:**
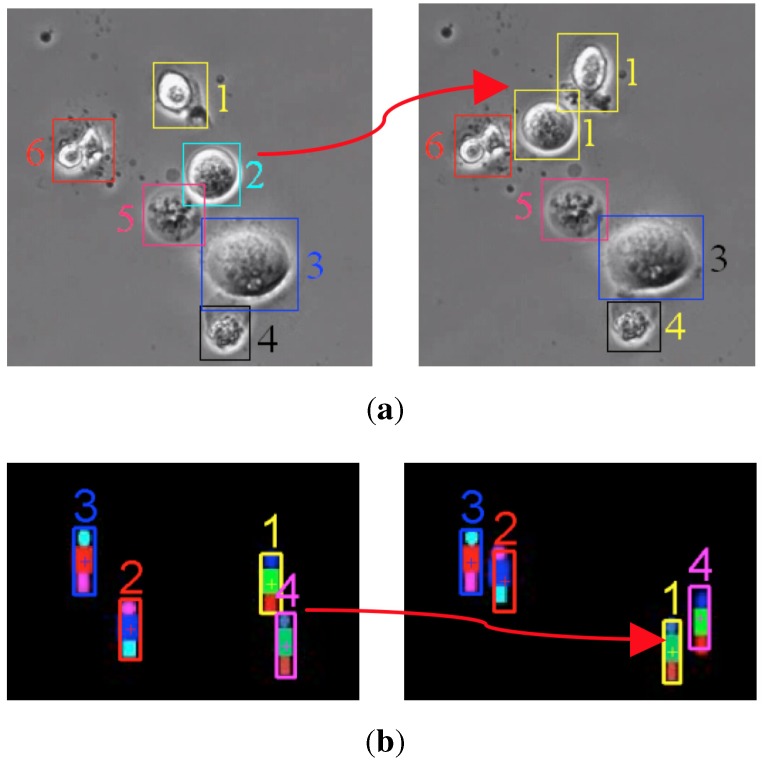
Tracking targets in close movement with the conventional Gaussian mixture probability hypothesis density (GM-PHD) tracker. (**a**) Mistracking two cells (Cells 1 and 2 in the left image) with the same identity (Cell 1 in the right image); (**b**) mistracking two targets (Targets 1 and 4 in the left image) with switched identities (Targets 4 and 1 in the right image).

Generally, each measurement is assumed to correspond to one target and *vice versa* in multi-target tracking. This so-called one-to-one assumption expresses that a target can only be associated with one measurement. However, in the GM-PHD tracker, this one-to-one assumption is violated whenever multiple measurements are close to one target. In other words, the efficiency of the GM-PHD tracker may degrade when targets come near each other. To remedy this, Yazdian-Dehkordi *et al.* proposed a competitive GM-PHD (CGM-PHD) tracker [20] and a penalized GM-PHD (PGM-PHD) tracker [21] to refine the weights of the close moving targets in the update step in the GM-PHD filter. However, they did not provide continuous trajectories for the targets. By considering this point, Wang *et al.* [22] proposed a collaborative penalized GM-PHD (CPGM-PHD) tracker, in which they utilized the track label of each Gaussian component to collaboratively penalize the weights of those close moving targets with the same identity. However, the aforementioned trackers are merely suitable for point target tracking, which may fail in video target tracking. Compared to the simple point representations of the target state and the measurement in point target tracking, the representations are more complicated in video target tracking. Both the location and the size of video targets are considered for modelling the target state and the measurement. As video targets move closely, the aforementioned trackers (GM-PHD tracker, CGM-PHD tracker, PGM-PHD tracker and CPGM-PHD tracker) may track multiple targets with the same identity (shown as in Figure 1a) or with switched identities (shown as in Figure 1b).

**Figure 2:**
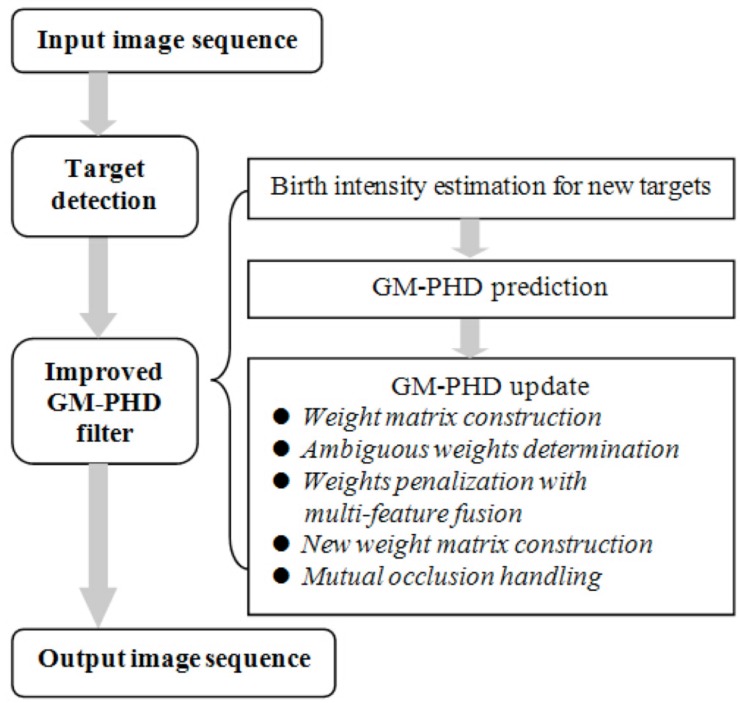
Pipeline of the proposed tracker.

As targets move close enough, mutual occlusion may occur. As a result, the measurements originating from targets within the occlusion region will be merged into one measurement. Without an occlusion handling method, the tracker may fail to track them. Because occlusion handling is not the main contribution of this paper, we incorporate our previous reported game-theoretical method [23] into the tracker to solve the mutual occlusion problem. In this paper, we propose an improved GM-PHD tracker to robustly track targets in a video, especially to track targets in close movement. The pipeline of the proposed tracker is shown in Figure 2, and the main contributions are listed as follows.

(1) An improved GM-PHD tracker with multi-feature fusion-based weight penalization is proposed to effectively track targets in a video, especially to track the targets in close movement.

(2) A weight matrix of all updated weights is constructed, and an effective ambiguous weights determination method is proposed. The conventional trackers (the CGM-PHD, PGM-PHD and CPGM-PHD trackers) only consider the total weight for ambiguous weights determination, which is not applicable for Case 2. In contrast, we utilize the total weight and predicted target states to effectively determine the ambiguous weights for Case 1 and Case 2, respectively. In this paper, Case 1 is the case that one target is associated with multiple measurements; while Case 2 is the case that one target is associated with one incorrect measurement. More details of Case 1 and Case 2 are stated in Section 2.3.

(3) Multiple features that include spatial-colour appearance, histogram of oriented gradient and target area are fused and incorporated into the tracker to penalize the ambiguous weights. By doing so, the weights of the mismatched targets can be greatly reduced, and thus, the tracking accuracy is improved.

The rest of this paper is organized as follows. Section 2 simply introduces the GM-PHD filter and its drawbacks. Section 3 presents the proposed tracker in detail. Some experimental results are discussed in Section 4, followed by concluding remarks in Section 5.

## 2. Problem Formulation

### 2.1. Target State and Measurement Representation

For an input image sequence, the kinematic state of a target *i* at time *t* is denoted by $x_{t}^{i}=\left\{l_{t}^{i},v_{t}^{i},s_{t}^{i}\right\}$, where $l_{t}^{i}=\left\{l_{x,t}^{i},l_{y,t}^{i}\right\}$, $v_{t}^{i}=\left\{v_{x,t}^{i},v_{y,t}^{i}\right\}$ and $s_{t}^{i}=\left\{w_{t}^{i},h_{t}^{i}\right\}$ are the location, velocity and bounding box size of the target, respectively; $i=1,\cdots ,N_{t}$, and $N_{t}$ denotes the number of targets at time *t*. The measurement originating from a target *j* at time *t* is denoted by $z_{t}^{j}=\left\{l_{z,t}^{j},s_{z,t}^{j}\right\}$, where $j=1,\cdots ,N_{m,t}$, and $N_{m,t}$ denotes the number of measurements at time *t*. The target state set and measurement set at time *t* are denoted by $X_{t}=\left\{x_{t}^{1},\cdots ,x_{t}^{N_{t}}\right\}$ and $Z_{t}=\left\{z_{t}^{1},\cdots ,z_{t}^{N_{m,t}}\right\}$, respectively. The measurements are obtained by object detection, and any object detection method can be used in our tracker. To show the robust performance of the proposed tracker, a simple background subtraction algorithm [15] is utilized to obtain the measurements.

### 2.2. The GM-PHD Filter

The GM-PHD filter was first proposed by Vo and Ma [7] in 2006. It is a closed-form solution to the PHD filter recursion, whose posterior intensity function is estimated by a sum of weighted Gaussian components that can be propagated analytically in time. More details of the GM-PHD filter are in the literature [7]. Generally, the GM-PHD filter can be implemented in the prediction and update steps.

Step 1: Prediction. Suppose that PHD $D_{t-1}\left(x_{t-1}\right)$ at time t-1 has the form $D_{t-1}\left(x_{t-1}\right)=\sum_{i=1}^{J_{t-1}}\omega_{t-1}^{\left(i\right)}N\left(x_{t-1};m_{t-1}^{\left(i\right)},P_{t-1}^{\left(i\right)}\right)$, then the predicted PHD $D_{t|t-1}\left(x_{t}\right)$ is given by:

(1) $$D_{t|t-1}\left(x_{t}\right)=\gamma_{t}\left(x_{t}\right)+p_{sv}\sum_{i=1}^{J_{t-1}}\omega_{t-1}^{\left(i\right)}N\left(x_{t};m_{sv,t|t-1}^{\left(i\right)},P_{sv,t|t-1}^{\left(i\right)}\right)$$

where $m_{sv,t|t-1}^{\left(i\right)}=F_{t-1}m_{t-1}^{\left(i\right)}$ and $P_{sv,t|t-1}^{\left(i\right)}=Q_{t-1}+F_{t-1}P_{t-1}^{\left(i\right)}F_{t-1}^{T}$. $\gamma_{t}\left(x_{t}\right)$ and $p_{sv}$ denote the probabilities of newborn targets and survival targets, respectively. N(·;m,P) denotes a Gaussian component with the mean m and covariance P. $F_{t-1}$ is the motion transition matrix.

Step 2: Update. The predicted PHD can be expressed as a Gaussian mixture $D_{t|t-1}\left(x_{t}\right)=\sum_{i=1}^{J_{t|t-1}}\omega_{t|t-1}^{\left(i\right)}N\left(x_{t};m_{t|t-1}^{\left(i\right)},P_{t|t-1}^{\left(i\right)}\right)$, then the posterior PHD $D_{t}\left(x_{t}\right)$ at time *t* is given by:

(2) $$D_{t}\left(x_{t}\right)=\left(1-p_{d}\right)D_{t|t-1}\left(x_{t}\right)+\sum_{z_{t}\in Z_{t}}D_{g,t}\left(x_{t};z_{t}\right)$$

(3) $$D_{g,t}\left(x_{t};z_{t}\right)=\sum_{i=1}^{J_{t|t-1}}\omega_{g,t}^{\left(i\right)}\left(z_{t}\right)N\left(x_{t};m_{g,t}^{\left(i\right)}\left(z_{t}\right),P_{g,t}^{\left(i\right)}\left(z_{t}\right)\right)$$

(4) $$\omega_{g,t}^{\left(i\right)}\left(z_{t}\right)=\frac{p_{d}\omega_{t|t-1}^{\left(i\right)}N\left(z_{t};m_{h,t}^{\left(i\right)},P_{h,t}^{\left(i\right)}\right)}{\lambda_{t}c_{t}\left(z_{t}\right)+p_{d}\sum_{i=1}^{J_{t|t-1}}\omega_{t|t-1}^{\left(i\right)}N\left(z_{t};m_{h,t}^{\left(i\right)},P_{h,t}^{\left(i\right)}\right)}$$

where $m_{g,t}^{\left(i\right)}\left(z_{t}\right)=m_{t|t-1}^{\left(i\right)}+K\left(z_{t}-H_{t}m_{t|t-1}^{\left(i\right)}\right)$, $K=P_{t|t-1}^{\left(i\right)}H_{t}^{T}{\left(H_{t}P_{t|t-1}^{\left(i\right)}H_{t}^{T}+R_{t}\right)}^{-1}$, $P_{g,t}^{\left(i\right)}\left(z_{t}\right)=\left(I-KH_{t}\right)P_{t|t-1}^{\left(i\right)}$, $m_{h,t}^{\left(i\right)}=H_{t}m_{t|t-1}^{\left(i\right)}$, $P_{h,t}^{\left(i\right)}=H_{t}P_{t|t-1}^{\left(i\right)}H_{t}^{T}+R_{t}$. $p_{d}$ is the detection probability. $\lambda_{t}$ and $c_{t}\left(z_{t}\right)$ are the average rate and probability density of the spatial distribution of Poisson distributed clutters, respectively. $H_{t}$ and $R_{t}$ are the measurement matrix and the covariance matrix of the measurement noise, respectively.

To predict the newborn targets, we need to find the peak (the mean of Gaussian) of intensity $\gamma_{t}\left(x_{t}\right)$, *i.e.*, the position where the targets are most probable to appear. To automatically and accurately estimate the birth intensity, our previous work [15] is utilized in this paper. Furthermore, we employ the pruning and merging algorithms [7] to prune the irrelevant components and to merge the same intensity components into one component. The peaks of the intensity are the points of the highest local concentration of the expected number $N_{t}$ of targets. Finally, we can estimate the target states with $N_{t}$ ordered mean with the largest weights.

### 2.3. Drawbacks of the GM-PHD Filter

**Figure 3:**
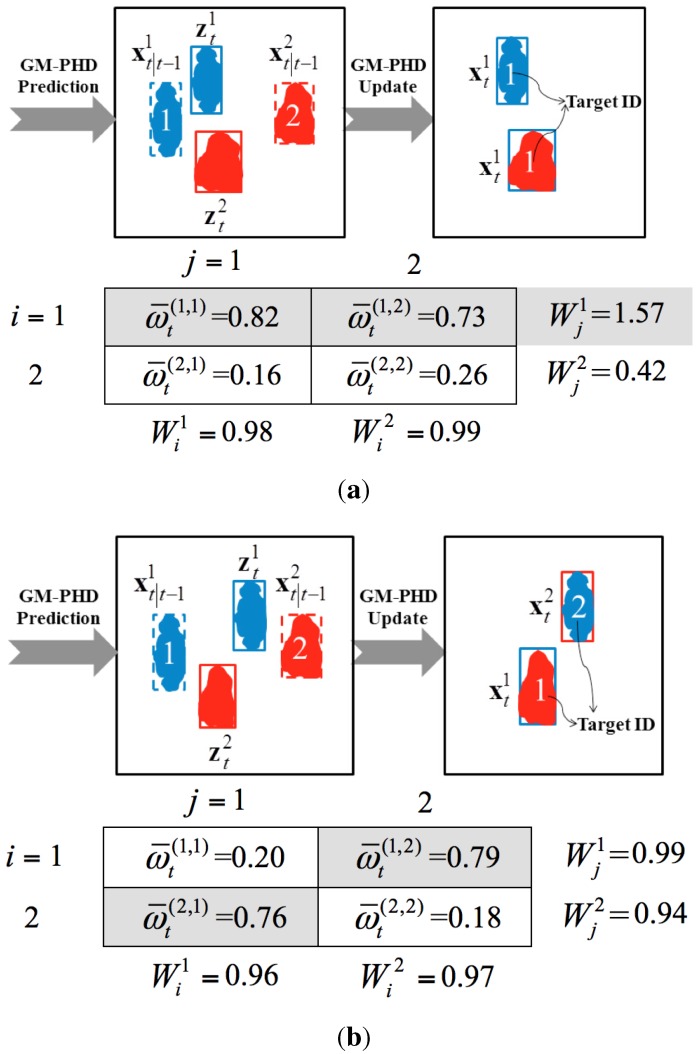
A pictorial example of tracking two targets in close movement. (**a**) Case 1: two targets with the same identity; (**b**) Case 2: two targets with switched identities.

The GM-PHD filter recursively propagates the first-order moment associated with the multi-target posterior density to avoid the complicated data association problem and, consequently, can be efficiently used in multiple video targets’ tracking. However, as targets come near each other, multiple measurements may associate with one target or incorrect targets. Normally, each predicted state $x_{t|t-1}^{\left(i\right)}$ of target *i* is associated with only one measurement $z_{t}^{j}$ originating from target *i*, which means that the weight of the *i*-th predicted target updated by the *j*t-h measurement should be far greater than those weights updated by other measurements. However, in the real-world scenarios, two possible cases could violate this one-to-one association. As a result, the GM-PHD filter may track multiple targets with the same identity or with switched identities. Figure 3 is a pictorial example of the aforementioned two cases when tracking two targets in close movement.

Case 1: One predicted target (shown as target $x_{t|t-1}^{1}$ in Figure 3a) may be associated with more than one measurement (shown as the measurements $z_{t}^{1}$ and $z_{t}^{2}$ in Figure 3a). In such a case, there are at least two updated weights for the same target (shown as ${\bar{\omega }}_{t}^{\left(1,1\right)}$ and ${\bar{\omega }}_{t}^{\left(1,2\right)}$ in Figure 3a), whose values are far greater than other updated weights. ${\bar{\omega }}_{t}^{\left(i,j\right)}$ is the normalized weight of target *i* updated by measurement *j*. For simplicity, indices *i* and *j* are used to represent the *i*-th predicted target state $x_{t|t-1}^{i}$ and the *j*-th measurement $z_{t}^{j}$, respectively. As a result, the GM-PHD tracker tracks Targets 1 and 2 with the same Identity 1 (shown as the right image in Figure 3a).

Case 2: one predicted target may be associated with another measurement that is not originated from this target. As shown in Figure 3b, measurement $z_{t}^{1}$ should theoretically be associated with Target 1, while measurement $z_{t}^{2}$ should be associated with Target 2. However, ${\bar{\omega }}_{t}^{\left(1,2\right)}$ is actually greater than ${\bar{\omega }}_{t}^{\left(1,1\right)}$, while ${\bar{\omega }}_{t}^{\left(2,1\right)}$ is greater than ${\bar{\omega }}_{t}^{\left(2,2\right)}$. As a result, the GM-PHD tracker tracks Targets 1 and 2 with switched Identities 2 and 1, respectively (shown as the right image in Figure 3b).

To improve the aforementioned drawbacks, an improved GM-PHD tracker with weight penalization is proposed.

## 3. Improved GM-PHD Tracker with Weight Penalization

The way of improving the drawbacks is to penalize the weights of those targets that move closely. First, a weight matrix that consists of all updated weights is constructed. Then, an ambiguous weight is defined, and the corresponding methods for searching ambiguous weights are proposed. Finally, multiple features are fused and incorporated into the tracker to penalize the ambiguous weights.

### 3.1. Weight Matrix Construction

**Figure 4:**
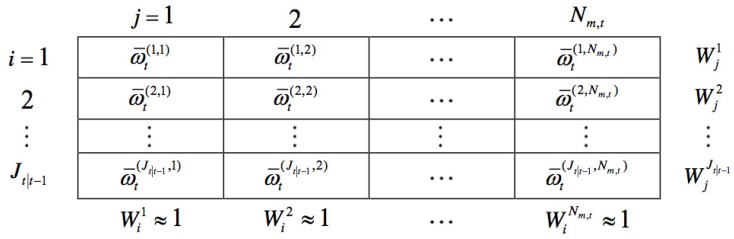
Weight matrix: a symbolic representation of updated weights.

Figure 4 is a symbolic representation of updated weights. For better clarification, the matrix that includes the weights of all targets updated by all measurements is called the weight matrix (shown as in Figure 4). In the weight matrix, the *i*-th row represents the weights of the *i*-th predicted target updated by all measurements, while the *j*-th column represents the weights of all predicted targets updated by the *j*-th measurement. $W_{j}^{i}=\sum_{j=1}^{N_{m,t}}{\bar{\omega }}_{t}^{\left(i,j\right)}$ in the figure is the total weight of the *i*-th row, while $W_{i}^{j}=\sum_{i=1}^{J_{t|t-1}}{\bar{\omega }}_{t}^{\left(i,j\right)}$ is the total weight of the *j*-th column. $N_{m,t}$ and $J_{t|t-1}$ are the numbers of measurements and predicted target states, respectively.

(5) $${\bar{\omega }}_{t}^{\left(i,j\right)}=\frac{p_{d}\omega_{t|t-1}^{\left(i\right)}N\left(z_{t}^{j};m_{h,t}^{\left(i\right)},P_{h,t}^{\left(i\right)}\right)}{\lambda_{t}c_{t}\left(z_{t}^{j}\right)+p_{d}\sum_{i=1}^{J_{t|t-1}}\omega_{t|t-1}^{\left(i\right)}N\left(z_{t}^{j};m_{h,t}^{\left(i\right)},P_{h,t}^{\left(i\right)}\right)}$$

### 3.2. Ambiguous Weight Determination

As stated in Section 2.2, the peaks of the updated GM-PHD are the points of the highest local concentration of the expected number $N_{t}$ of targets. However, an incorrect estimate of the multi-target state may be obtained when targets move in a close space (as explained in the cases listed in Section 2.3). To remedy this, the incorrect weights should be penalized. In this paper, the weights of those close moving targets are defined as the ambiguous weights. Before penalization, the weight matrix should be analysed first to determine the ambiguous weights. In the CGM-PHD tracker [20], PGM-PHD tracker [21] and CPGM-PHD tracker [22], the weight of target *i* is determined as an ambiguous weight once the total weight $W_{j}^{i}$ of the *i*-th row is greater than one. However, this method is not applicable to Case 2 (as stated in Section 2.3) since the total weight $W_{j}^{i}$ may be less than one when targets approach each other. To remedy this, both the total weight $W_{j}^{i}$ and the predicted target states are utilized to determine the ambiguous weights of Case 1 and Case 2, respectively.

(1) Ambiguous weights’ determination for Case 1 

Normally, as targets are all correctly associated, the total weights $W_{j}^{i}$ should be approximate to one according to Equation (5). However, when targets move closely and simultaneously, multiple measurements are closer to one target *i* compared to the other targets; Gaussians in the *i*-th row in the weight matrix related to these measurements may have large enough weights. As a result, the total weights $W_{j}^{i}$ may be greater than one. In other words, for a given weight matrix, if the total weight $W_{j}^{i}$ of the *i*-th row satisfies the following condition:

(6) $$W_{j}^{i}>1$$

this weight matrix is determined as an ambiguous weight matrix. The ambiguous weight matrix shows the possibility that one or more ambiguous weights may be involved in this matrix. To further determine the ambiguous weights, the expected targets’ number and weight index in the matrix are used.

First, the expected number $N_{t}$ of targets is calculated according to the method proposed in Section 2.2.

Then, the first $N_{t}$ largest weights in the ambiguous weight matrix are selected as the ambiguous candidates.

Finally, if more than one candidate is in the same row in the matrix, these candidates are determined as the ambiguous weights. Otherwise, no ambiguous weights are involved. In other words, if more than one candidate has the same row index *i*, the corresponding weights ${\bar{\omega }}_{t}^{\left(i,j\right)}$ and ${\bar{\omega }}_{t}^{\left(i,j^{\prime }\right)}$ are determined as the ambiguous weights. $j^{\prime }\neq j$ and $j^{\prime }\in \left\{1,2,\cdots ,N_{m,t}\right\}$. The related measurements *j* and $j^{\prime }$ are determined as the ambiguous measurements, which are prone to be associated with the same target *i*. Consequently, the ambiguous weights ${\bar{\omega }}_{t}^{\left(i,j\right)}$ and ${\bar{\omega }}_{t}^{\left(i,j^{\prime }\right)}$ should be penalized. For example, the weights ${\bar{\omega }}_{t}^{\left(1,1\right)}$ and ${\bar{\omega }}_{t}^{\left(1,2\right)}$ in Figure 3a can be determined as the ambiguous weights according to the proposed method.

(2) Ambiguous weights’ determination for Case 2 

To determine the ambiguous weights for Case 2, those targets that move closely should be determined first. Targets *i* and $i^{\prime }$ are regarded as two close moving targets when:

(7) $$∥l_{t|t-1}^{i}-l_{t|t-1}^{i^{\prime }}∥<∥s_{t|t-1}^{i}∥+∥s_{t|t-1}^{i^{\prime }}∥$$

where $l_{t|t-1}^{i}$ (or $l_{t|t-1}^{i^{\prime }}$) and $s_{t|t-1}^{i}$ (or $s_{t|t-1}^{i^{\prime }}$) are the location and size of the predicted state $x_{t|t-1}^{i}$ (or $x_{t|t-1}^{i^{\prime }}$) of the target *i* (or $i^{\prime }$), respectively. $∥\cdot ∥$ represents the Euclidean norm.

Then, the ambiguous weights of Case 2 can be determined according to the measurements originating from the close moving targets. For two close moving targets *i* and $i^{\prime }$, if more than one measurement satisfies the following condition, these weights ${\bar{\omega }}_{t}^{\left(i,j\right)}$ can be regarded as the ambiguous weights.

(8) $$∥l_{z,t}^{j}-l_{t|t-1}^{i}∥<∥l_{t|t-1}^{i}-l_{t|t-1}^{i^{\prime }}∥$$

where $l_{z,t}^{j}$ is the location of the *j*-th measurement $z_{t}^{j}$.

After the ambiguous weights between the measurement *j* and the target *i* have been determined, multiple features that include the spatial-colour appearance, histogram of oriented gradient and target area are fused to penalize these ambiguous weights.

### 3.3. Multi-Feature Fusion

(1) Spatial-colour appearance 

A colour histogram of a target is a representation of the distribution of colours inside this target’s region in an image. Colour histogram-based appearances [24,25,26,27] are effective and efficient at capturing the distribution characteristics of visual features inside the target regions for visual tracking. In this section, a spatial constraint colour histogram appearance model (so-called spatial-colour appearance model) is presented.

**Figure 5:**
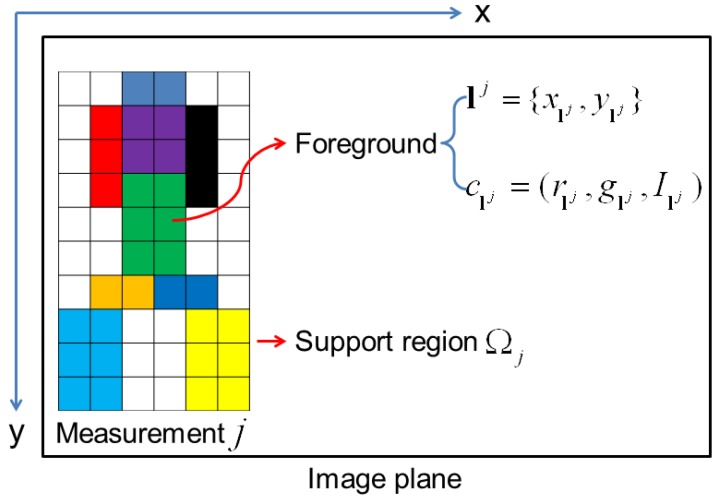
A schematic diagram of the colour distributions of the foreground pixels and the support region of the measurement *j*.

The appearance of a target *i* is modelled as a Gaussian mixture $q_{i}=q_{i}\left(\omega_{k}^{i},\mu_{k}^{i},\sum_{k}^{i}\right)$, representing the colour distribution of a target’s pixels [24]. k=1,⋯,K, and *K* is the number of Gaussian components. The measure of the similarity $P_{s}\left(i,j\right)$ between the measurement *j* and the target *i* is then defined by:

(9) $$P_{s}\left(i,j\right)=\text{exp}\left\{\frac{1}{N_{j}}\sum_{\Omega_{j}}\text{log}\left\{\sum_{k=1}^{K}\omega_{k}^{i}N\left(c_{l^{j}};\mu_{k}^{i},\sum_{k}^{i}\right)\right\}\right\}$$

(10) $$N\left(c;\mu ,\sum \right)=\frac{\text{exp}\left\{-\frac{1}{2}{\left(c-\mu \right)}^{\prime }\sum^{-1}\left(c-\mu \right)\right\}}{\sqrt{2\pi \left|\sum \right|}}$$

where $c_{l^{j}}=\left(r_{l^{j}},g_{l^{j}},I_{l^{j}}\right)$ is the colour of the pixel located in $l^{j}$ within the support region $\Omega_{j}$ of the measurement *j*. $N_{j}$ is the number of foreground pixels in $\Omega_{j}$. $g_{l^{j}}=G_{l^{j}}/\left(R_{l^{j}}+G_{l^{j}}+B_{l^{j}}\right)$, $r_{l^{j}}=R_{l^{j}}/\left(R_{l^{j}}+G_{l^{j}}+B_{l^{j}}\right)$ and $I_{l^{j}}=\left(R_{l^{j}}+G_{l^{j}}+B_{l^{j}}\right)/3$. Figure 5 is a schematic diagram of the colour distribution of the foreground pixels within a measurement’s region.

However, the aforementioned appearance model may fail when targets have similar colour distributions. To remedy this, a Gaussian spatial constraint [26] is incorporated, and the measure of the similarity is improved by:

(11) $$P_{s}\left(i,j\right)=\text{exp}\left\{\frac{1}{N_{j}}\sum_{\Omega_{j}}\text{log}\left\{N\left(l_{j};l_{t}^{i},\sum_{t}^{i}\right)\sum_{k=1}^{K}\omega_{k}^{i}N\left(c_{l^{j}};\mu_{k}^{i},\sum_{k}^{i}\right)\right\}\right\}$$

where $N(l_{j};l_{t}^{i},\sum_{t}^{i})$ is the Gaussian spatial constraint of the locations of the foreground pixels, and $\sum_{t}^{i}=\left[{\left(w_{t}^{i}/2\right)}^{2},0;0,{\left(h_{t}^{i}/2\right)}^{2}\right]$. $l_{t}^{i}=\left\{l_{x,t}^{i},l_{y,t}^{i}\right\}$ and $\left\{w_{t}^{i},h_{t}^{i}\right\}$ are the location and size of bounding box of the target *i* at time *t*, respectively.

(2) Histogram of oriented gradient [28] 

The gradient G(x,y) and orientation O(x,y) of each pixel in the target region is calculated by:

(12) $$G\left(x,y\right)=\sqrt{{\left[I\left(x+1,y\right)-I\left(x-1,y\right)\right]}^{2}+{\left[I\left(x,y+1\right)-I\left(x,y-1\right)\right]}^{2}}$$

(13) $$O\left(x,y\right)=\text{arctan}\left\{\frac{I(x,y+1)-I(x,y-1)}{I(x+1,y)-I(x-1,y)}\right\}$$

where I(x,y) is the location of pixel in the image *I*.

The weighted oriented gradient histogram $q_{h}^{i}\left(u\right)$ of target *i* is formed by dividing the orientation into 36 bins ($10^{\circ }$ each step).

(14) $$q_{h}^{i}\left(u\right)=C\sum_{r=1}^{n_{i}}k\left({∥(l_{r}^{i}-l_{0}^{i})/h∥}^{2}\right)G\left(l_{r}^{i}\right)\delta \left[b\left(l_{r}^{i}\right)-u\right]$$

where u=1,2,⋯,36, $C=1/\sum_{i=1}^{n_{i}}k\left({∥l_{r}^{i}∥}^{2}\right)$ is a normalization function, $n_{i}$ is the number of pixels in target *i*’s region, k(·) is an isotropic kernel profile, $l_{r}^{i}$ is the location of pixel *r*, *h* is the bandwidth, *δ* is the Kronecker delta function and $b(l_{r}^{i})$ associates the pixel *r* with the histogram bin.

The gradient of oriented histogram likelihood between the measurement *j* and the target *i* is defined by:

(15) $$P_{h}\left(i,j\right)=\frac{1}{\sqrt{2\pi }\sigma_{h}}\text{exp}\left\{\frac{-d_{h}^{2}\left[q_{h}^{i}\left(u\right),q_{h}^{j}\left(u\right)\right]}{2\sigma_{h}}\right\}$$

(16) $$d_{h}^{2}\left[q_{h}^{i}\left(u\right),q_{h}^{j}\left(u\right)\right]=\sqrt{1-\rho [q_{h}^{i}\left(u\right),q_{h}^{j}\left(u\right)]}$$

(17) $$\rho \left[q_{h}^{i}\left(u\right),q_{h}^{j}\left(u\right)\right]=\sum_{u=1}^{36}\sqrt{q_{h}^{i}\left(u\right)\cdot q_{h}^{j}\left(u\right)}$$

where $\sigma_{h}$ is the Gaussian variance, which is set as 0.3 in our experiments.

(3) Target area 

The degree of change between the areas of the target *i* and measurement *j* is defined by:

(18) $$P_{a}\left(i,j\right)=\frac{\text{min}\{S_{i},S_{j}\}}{\text{max}\{S_{i},S_{j}\}}$$

where $S_{i}$ and $S_{j}$ represent the areas of target *i* and measurement *j*, respectively. It is reasonable to state that the larger the $P_{a}\left(i,j\right)$ is, the more possible it is that the measurement *j* is generated from the target *i*, because the size of the same target changes slightly between two consecutive frames.

(4) Multi-feature fusion 

In this paper, the aforementioned features are fused to robustly penalize the ambiguous weight between the measurement *j* and the target *i*.

(19) $$P_{f}\left(i,j\right)=\left(P_{s}\left(i,j\right)+P_{h}\left(i,j\right)+P_{a}\left(i,j\right)\right)/3$$

Obviously, the larger the $P_{f}\left(i,j\right)$ is, the more possibility there is that the measurement *j* is generated from the target *i*. In fact, if a measurement *j* is truly generated from a target *i*, the $P_{f}\left(i,j\right)$ should approximately be one.

### 3.4. Weight Penalization

The ambiguous weight ${\bar{\omega }}_{t}^{\left(i,j\right)}$ can be penalized according to the multi-feature fusion.

(20) $${\bar{\omega }}_{t}^{\left(i,j\right)}={\bar{\omega }}_{t}^{\left(i,j\right)}\cdot P_{f}\left(i,j\right)$$

After all of the ambiguous weights have been penalized, all of the weights in the *j*-th column in the weight matrix should be further normalized by:

(21) $${\bar{\omega }}_{t}^{\left(i,j\right)}={\bar{\omega }}_{t}^{\left(i,j\right)}/W_{i}^{j}$$

where $i=1,\cdots ,J_{t|t-1}$.

## 4. Experimental Evaluation

Our tracker can be employed for various scenarios, such as person tracking for human behaviour surveillance and analysis, car tracking for traffic surveillance, human hand and object tracking for human-object interactions, cell tracking for biomedical application, and so on.

In this section, we first evaluate our weight penalization method on several kinds of scenarios that include synthetic image sequences, outdoor human surveillance and cell moving surveillance scenarios by comparing to the state-of-the-art weight penalization methods. We then qualitatively test the proposed tracker on three more challenging scenarios and quantitatively compare it to several state-of-the-art trackers.

To quantitatively evaluate the tracking performance, the CLEAR MOTmetrics [23] is used. This returns a precision score MOTP (multi-object tracking precision) and an accuracy score MOTA (multi-object tracking accuracy) that is composed of a miss rate (MR), a false positive rate (FPR) and a mismatch rate (MMR).

(22) $$\text{MOTP}=\frac{\sum_{i,t}\left[S\left(gb_{t}^{i}\cap tb_{t}^{i}\right)/S\left(gb_{t}^{i}\cup tb_{t}^{i}\right)\right]}{\sum_{t}c_{t}}$$

(23) $$\text{MOTA}=1-\frac{\sum_{t}\left(m_{t}+fp_{t}+mme_{t}\right)}{\sum_{t}g_{t}}$$

where S(·) represents the area. $gb_{t}^{i}$ is the ground truth box, and $tb_{t}^{i}$ is the associated tracked box of the target *i* for time *t*. $c_{t}$ is the number of matched targets for time *t*. $m_{t}$, $fp_{t}$, $mme_{t}$ and $g_{t}$ are the numbers of misses, false positives, mismatches and ground truths, respectively, for time *t*.

### 4.1. Experimental Parameter Setup

Parameters of the tracker involved in the experiments are set as follows. Similarly as set in our previous work [15], we have the state transition model as $F_{t}=\left[I_{2},TI_{2},0_{2};0_{2},I_{2},0_{2};0_{2},0_{2},I_{2}\right]$ and $Q_{t}=\delta_{v}^{2}\left[T^{4}I_{2}/4,T^{3}I_{2}/2,0_{2};T^{3}I_{2}/2,T^{2}I_{2},0_{2};0_{2},0_{2},T^{2}I_{2}\right]$, where $0_{n}$ and $I_{n}$ are the n×n zero and identity matrices. *T* = 1 frame is the interval between two consecutive time steps. $\delta_{v}$ = 3 is the standard deviation of the state noise. We also set the measurement model as $H_{t}=\left[I_{2},0_{2},0_{2};0_{2},0_{2},I_{2}\right]$ and $R_{t}=\delta_{w}^{2}I_{4}$, where $\delta_{w}$ = 2 is the standard deviation of the measurement noise. The values of residual parameters involved in our tracker are set as: $p_{d}$ = 0.99, $p_{sv}$ = 0.95, $\lambda_{t}$ = 0.01, $c_{t}\left(z_{t}\right)$ = (image area)^−1^ and $\sigma_{h}$ = 0.3.

### 4.2. Evaluation of the Proposed Weight Penalization Method

We evaluate the proposed weight penalization method on three scenarios, including a synthetic image sequence, an outdoor human surveillance scenario and a cell moving surveillance scenario. Moreover, to demonstrate the effectiveness of the proposed method, it is also compared to the conventional GM-PHD tracker [7] and the CPGM-PHD tracker [22].

(1) Qualitative analysis 

**Figure 6:**
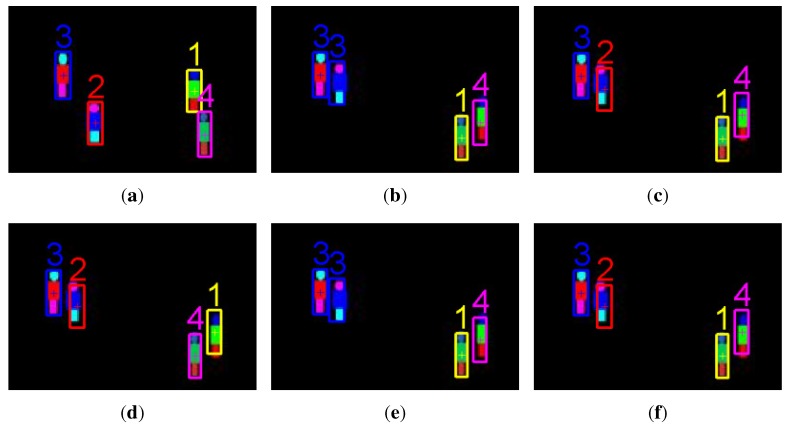
Tracking results on a synthetic image sequence. (**a**) Tracked targets at *t* = 48 by all of the trackers; (**b**) tracked targets at *t* = 49 by the GM-PHD tracker; (**c**) tracked targets at *t* = 49 by the collaborative penalized GM (CPGM)-PHD tracker; (**d**) tracked targets at *t* = 49 by our tracker with multi-feature fusion; (**e**) tracked targets at *t* = 49 by our tracker with the target area feature; (**f**) tracked targets at *t* = 49 by our tracker with the colour appearance feature.

**Figure 7:**
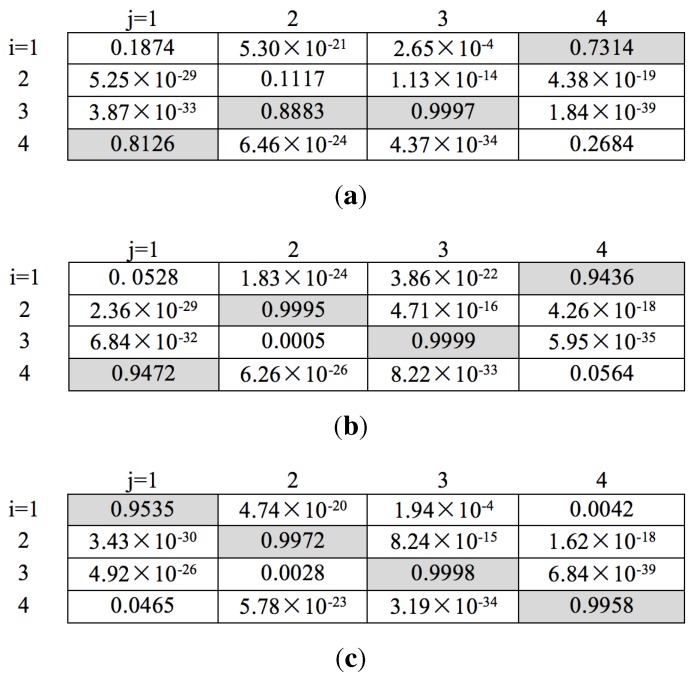
Updated weight matrices at *t* = 49 on a synthetic image sequence. (**a**) For the GM-PHD tracker; (**b**) for the CPGM-PHD tracker; (**c**) for our tracker.

**Figure 8:**
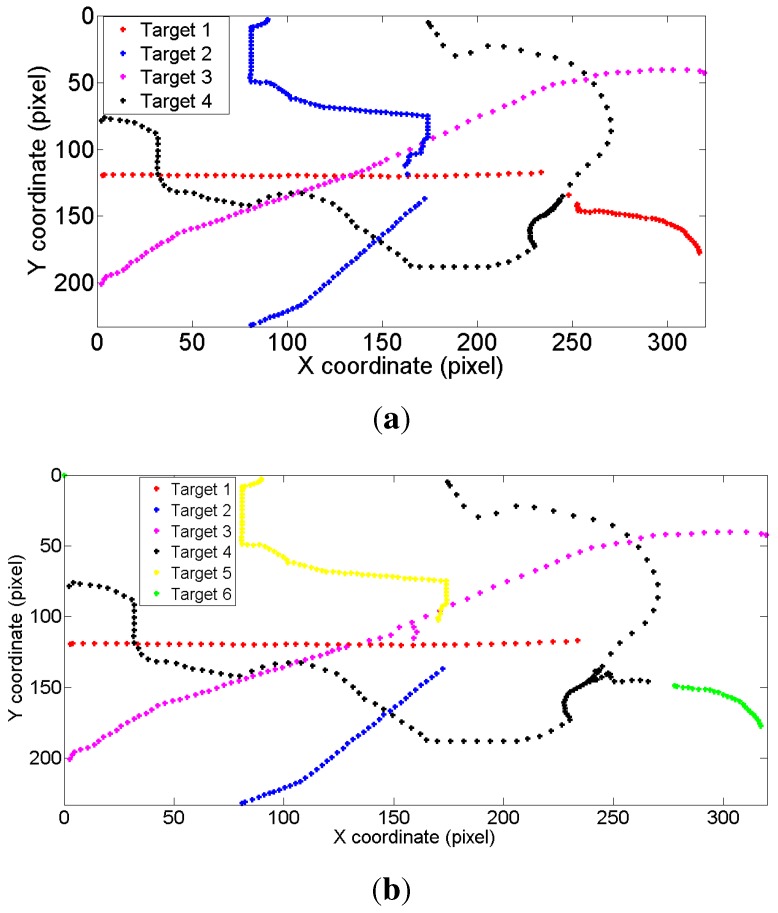

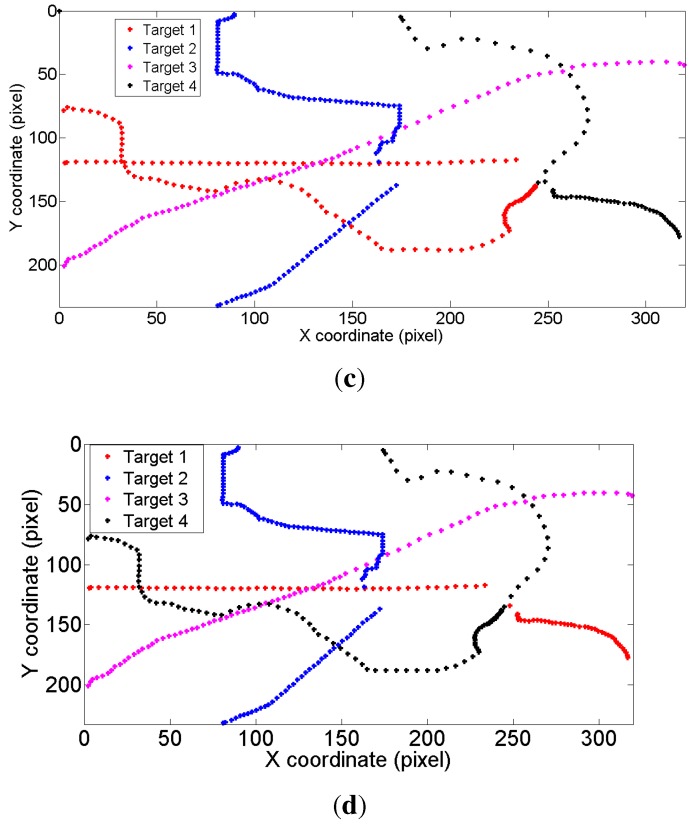
Tracking trajectories on a synthetic image sequence. (**a**) Ground truth; (**b**) for the GM-PHD tracker; (**c**) for the CPGM-PHD tracker; (**d**) for our tracker.

Tracking on a synthetic image sequence: A synthetic image sequence is used to validate the effectiveness of the proposed weight penalization method. Figure 6 and Figure 7 show the tracking results and the corresponding weight matrices obtained by the trackers, respectively. At *t* = 48, all of the trackers can successfully track all of the targets (shown as in Figure 6a). At *t* = 49, Targets 1 and 4 approach very close, as well as Targets 2 and 3. Without any weight penalization method, the conventional GM-PHD tracker tracks Target 2 with the wrong Identity 3, while switching the identities for Targets 1 and 4 (shown as in Figure 6b). According to the method proposed in the CPGM-PHD tracker, two ambiguous weights for Case 1 are determined and rearranged (shown as in Figure 7b), and the corresponding targets are tracked with correct identities (shown as Targets 2 and 3 in Figure 6c). However, the CPGM-PHD tracker cannot correctly track the targets with switched identities for Case 2 (shown as Targets 1 and 4 in Figure 6c). On the contrary, our tracker determines the ambiguous weights for both Case 1 and Case 2 and penalizes the ambiguous weights by fusing the multiple target features. By doing so, four ambiguous weights are determined to be rearranged (shown as in Figure 7c), and all of the targets are tracked with correct identities (shown as in Figure 6d). Figure 8 shows the trajectories of the tracked targets. The results demonstrate that the trajectories obtained by our tracker are closer to the ground truth.

To show the effectiveness of multi-feature fusion, we also perform our tracker with a single feature, such as the target area feature and the colour appearance feature. The tracking results are shown as in Figure 6e,f. Since the target areas of two closely moving targets (Targets 2 and 3) are almost the same, the difference between the measurements originating from them is negligible. If we only use target area to penalize the weights, the tracker should perform just like a conventional GM-PHD tracker (shown as Figure 6e). Although the areas of Targets 2 and 3 are almost the same, their appearances are totally different. Therefore, penalizing the weights with the colour appearance feature can correctly track these two targets. However, many similarities occur in Targets 1 and 4, which results in a mismatched result.

**Figure 9:**
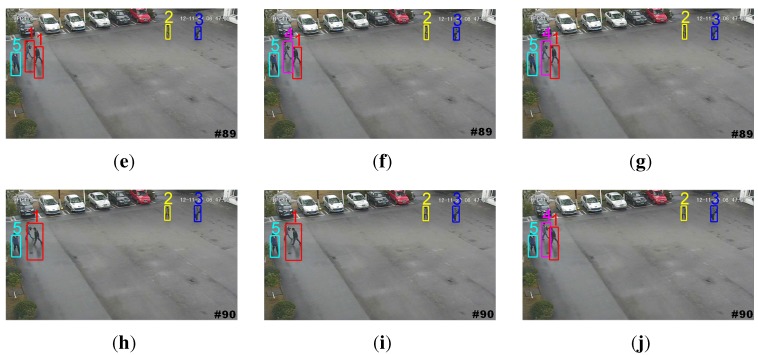
Tracking results on an outdoor human surveillance scenario. (**a**,**d**) Tracked targets by the GM-PHD tracker; (**b**,**e**) tracked targets by the CPGM-PHD tracker; (**c**,**f**) tracked targets by our tracker.

**Figure 10:**
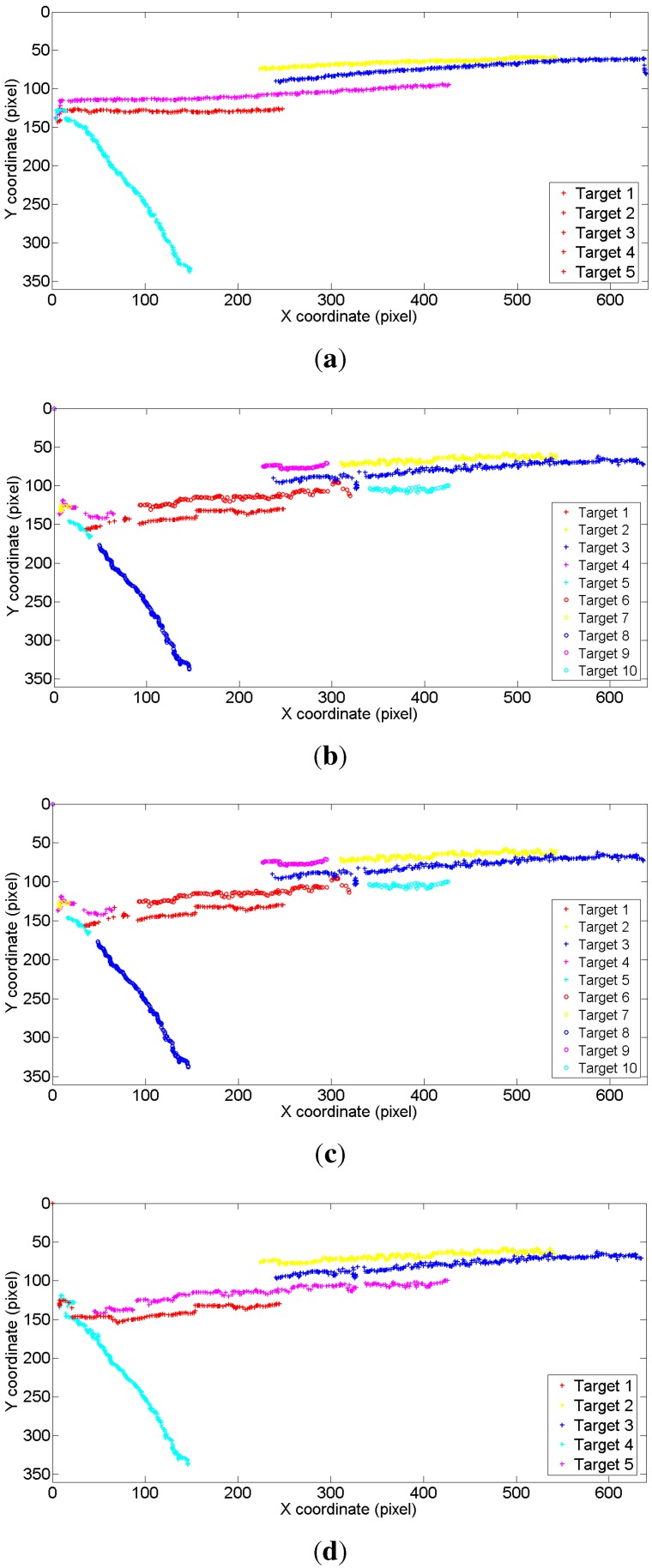
Tracking trajectories on an outdoor human surveillance scenario. (**a**) Ground truth; (**b**) for the GM-PHD tracker; (**c**) for the CPGM-PHD tracker; (**d**) for our tracker.

Tracking on an outdoor human surveillance scenario: An outdoor human surveillance sequence is used to further evaluate the proposed weight penalization method. Figure 9 shows the tracking results by the GM-PHD tracker, the CPGM-PHD tracker and our tracker, respectively. Without the weight penalization method, the conventional GM-PHD tracker tracks the close moving targets with the same identities at *t* = 89 (shown as two Target 1s in Figure 9a). Both the CPGM-PHD and proposed trackers can successfully track the closely moving targets at *t* = 89 (shown as in Figure 9b,c). However, both of the GM-PHD and CPGM-PHD trackers track the merged measurement as one single target, as mutual occlusion occurs in targets at *t* = 90 (shown as Target 1 in Figure 9d,e). On the contrary, our tracker can correctly track the targets in mutual occlusion (shown as Targets 1 and 5 in Figure 9f) by incorporating the mutual occlusion handling method. Figure 10 demonstrates the trajectories of the tracked targets. The GM-PHD tracker and CPGM-PHD tracker cannot correctly track the targets in consecutive time steps and, thus, results in many mismatches. By contrast, the results obtained by our tracker are closer to the ground truth and, thus, largely reduce the mismatches.

**Figure 11:**
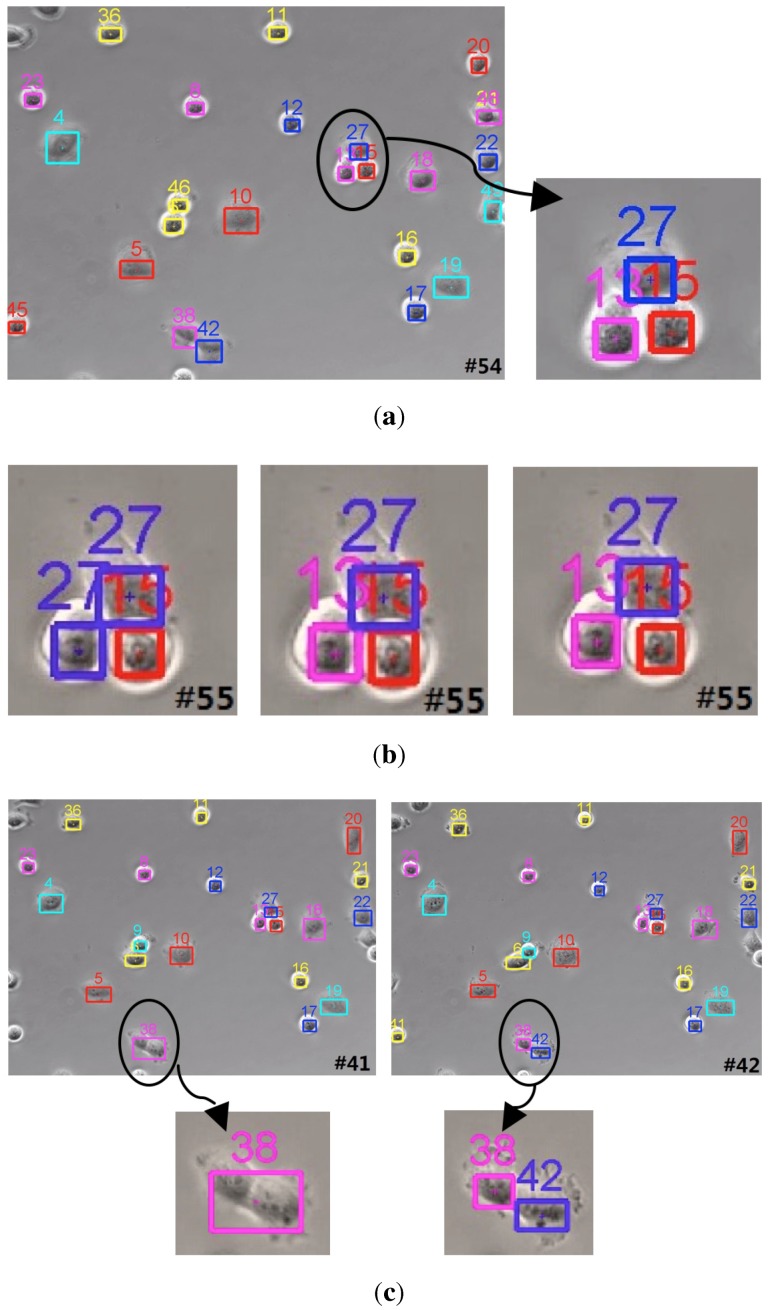
Tracking results on a cell moving surveillance scenario. (**a**) Left: tracked targets at *t* = 54; right: local tracked targets; (**b**) from left to right: tracked targets at *t* = 55 by the GM-PHD tracker, by the CPGM-PHD tracker and by our tracker, respectively; (**c**) from left to right: tracked targets by our tracker at *t* = 41 and *t* = 42, respectively.

Tracking on a cell moving surveillance scenario: A cell moving surveillance sequence captured from the phase contrast microscopy video is tested to evaluate the robustness of the proposed tracker. The high density of the cell population makes cells move in a relatively close space. Without weight penalization, the GM-PHD tracker may track the closely moving cells with the same identity. As shown in the left image in Figure 11b, two cells are assigned with the same ID 27. On the contrary, both trackers (the CPGM-PHD tracker and the proposed tracker) with the weight penalization method can successfully track the cells with the correct identities (shown as the middle and right images in Figure 11b). Nevertheless, our tracker can achieve more exact cell states. Moreover, our tracker can successfully track the mitosis cell as a newborn cell (shown as in Figure 11c) because of the incorporation of an effective birth intensity estimation method.

(2) Quantitative analysis 

We quantitatively evaluate the tracking performance according to the CLEAR MOT metrics. Table 1 shows the corresponding tracking performance comparison of the GM-PHD tracker, CPGM-PHD tracker and our tracker tested on the above-mentioned surveillance scenarios. The results show that tracking with our tracker can achieve better scores, both in MOTP and MOTA, on the tested sequences. Moreover, to show the effectiveness of the proposed weight penalization method for tracking the closely moving targets, the mismatch rate is also compared (shown as in Table 2). By determining the ambiguous weights for two cases and incorporating multiple target features to penalize the ambiguous weights, our tracker can reduce the mismatch rate and, thus, improve the tracking accuracy.

**Table 1:** Tracking performance comparison of the GM-PHD tracker, CPGM-PHD tracker and our tracker. MOTA, multi-object tracking accuracy; MOTP, multi-object tracking precision.

Tracker	Performance	Synthetic Images	Outdoor Human Surveillance	Cells Moving
GM-PHD	MOTA	0.8586	0.6265	0.5128
tracker [7]	MOTP	0.9266	0.8567	0.4283
CPGM-PHD	MOTA	0.9863	0.7038	0.6842
tracker [22]	MOTP	0.9536	0.8724	0.5581
Our	MOTA	1	0.9348	0.7218
tracker	MOTP	0.9675	0.9273	0.6065

**Table 2:** Mismatch rate (MMR: %) comparison of the GM-PHD tracker, CPGM-PHD tracker and our tracker.

Tracker	Synthetic Images	Outdoor Human Surveillance	Cells Moving
GM-PHD tracker	12.88	8.52	18.75
CPGM-PHD tracker	1.37	2.76	7.13
Our tracker	0	0.94	2.68

### 4.3. Evaluation of the Proposed Tracker

We first qualitatively evaluate our tracker on three more challenging surveillance scenarios, including interactive person tracking for public surveillance, person and luggages tracking for subway station surveillance of PETS2006 [29] and crowd person tracking for campus surveillance of PETS2009 [30], and then quantitatively compare our tracker with the state-of-the-art trackers according to the CLEAR MOT metrics. Moreover, the computational cost of our tracker on tested surveillance scenarios is also presented and discussed.

(1) Qualitative and quantitative analysis 

**Figure 12:**
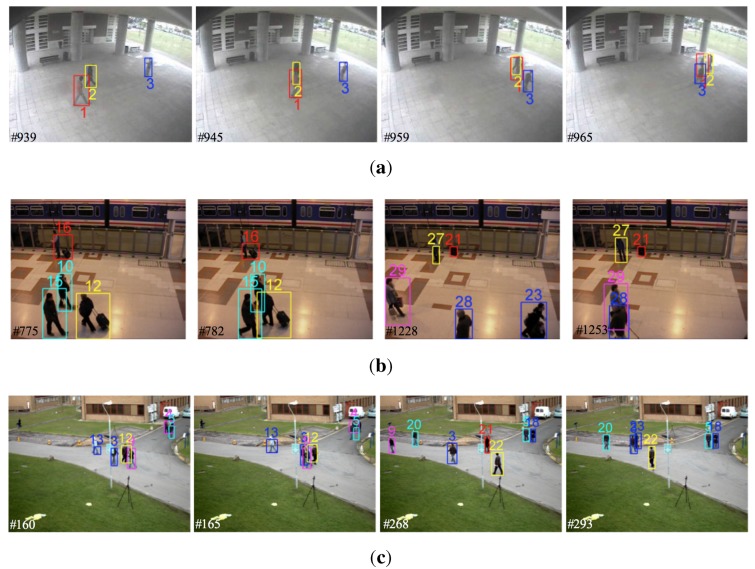
Tracking results of our tracker on three challenging surveillance scenarios. (**a**) Tracking interactive persons for public surveillance; (**b**) tracking persons and luggage for subway station surveillance; (**c**) tracking crowd persons for campus surveillance.

Figure 12 shows tracking results of our tracker tested on the above-mentioned three challenging surveillance scenarios. In Figure 12a, three persons move closely and frequently interact with each other. At *t* = 939, Person 1 and Person 2 get close, and occlusion occurs at *t* = 945. At *t* = 959, three persons get close, and a long-term occlusion occurs among them. Although persons get close and interact frequently, our tracker can successfully track all three persons with correct identities with time due to the effective weight penalization and occlusion handling method. In Figure 12b, three persons get close at *t* = 775, and serious occlusion occurs with almost the same appearance at *t* = 782. With our multi-feature fusion scheme, as well as occlusion handling method, the persons involved are accurately tracked. It is noted that when a person is moving with luggage in his or her hand, both the person and luggage are tracked as one single target (shown as Targets 12 and 16 in Figure 12b). However, when they are separated, they are tracked as two targets with different identities (shown as Targets 21 and 27 in Figure 12b). In Figure 12c, a large number of persons and many interactions are involved. For example, at *t* = 160 and *t* = 165, Persons 3, 9 and 12, as well as Persons 4 and 5 are walking together. Similarly, at *t* = 293, Persons 3 and 23, as well as Persons 5 and 18 get close, and occlusions occur. Even though, our tracker still can effectively track those persons with correct identities.

To show the superior performance of our tracker, it is quantitatively compared to the state-of-the-art trackers according to the CLEAR MOT metrics. We compare the MOTA and MOTP scores of our tracker with the scores reported in [31,32,33] on the subway station surveillance scenario of PETS2006, and the scores reported in [34,35,36] on the campus surveillance scenario of PETS2009, respectively. The results in Table 3 and Table 4 show that our tracker achieves a better MOTP score on tracking precision and a comparable MOTA score on tracking accuracy. The reason for the lower MOTA score than the score reported in [35] is that we implement object detection using a simple background subtraction method. This simple method tends to generate a large number of noise in variable environment. Although our tracker can eliminate a large number of noise, some noises may still be tracked as the targets. To further achieve a high MOTA score, a more robust object detection method should be incorporated.

**Table 3:** Tracking performance comparison on the subway station surveillance scenario of PETS2006.

	GM-PHD Tracker [7]	Tracker in [31]	Tracker in [32]	Tracker in [33]	Our Tracker
MOTA	0.3440	0.9875	0.9221	0.9656	0.8861
MOTP	0.4286	0.5816	0.4980	0.5687	0.6346

**Table 4:** Tracking performance comparison on the campus surveillance scenario of PETS2009.

	GM-PHD Tracker [7]	Tracker in [34]	Tracker in [35]	Tracker in [36]	Our Tracker
MOTA	0.4617	0.7591	0.8932	0.7977	0.8826
MOTP	0.4976	0.5382	0.5643	0.5634	0.6055

(2) Computational cost 

**Figure 13:**
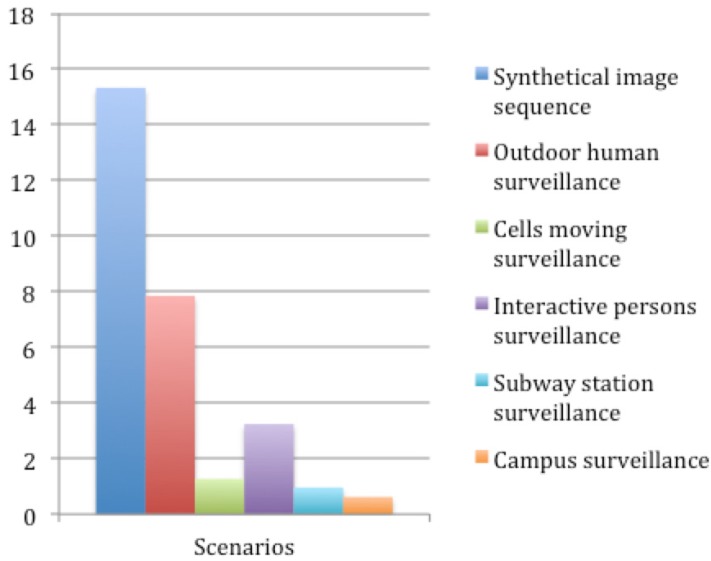
Processing time (unit: fps) of our tracker on the tested scenarios.

The proposed tracker is implemented in MATLAB using a computer with Inter(R) Core(TM) i7-4600U CPU 2.10 GHz and 4 GB of memory. Without any code optimization, the average runtimes of the tested surveillance videos are shown as in Figure 13. The majority of the runtimes are consumed in game theory-based mutual occlusion handling, because it is a pixel-wise iteration process. In addition, tracking a larger number of targets also increases the computational burden. In cell moving surveillance, subway station surveillance and campus surveillance scenarios, a large number of targets, as well as many occlusions are involved, which cost more computational time and slow down the processing speed.

## 5. Conclusions

We have developed a robust GM-PHD tracker to track targets in close movement in video. We incorporated an entropy-based birth intensity estimation method to effectively eliminate the false positives caused by noises. Particularly, we presented a weight penalization method to accurately track the targets in close movement.

The majority of the leading methods in the state of the art only considered ambiguous weight penalization for Case 1. Besides, only the total weight was used for ambiguous weight determination. However, both Case 1 and Case 2 could cause incorrect tracking. In this paper, we constructed a weight matrix and used both the total weight and target state to determine the ambiguous weights for both cases in the matrix. We then fused multiple target features, including the spatial-colour appearance, histogram of oriented gradient and target area, to penalize the ambiguous weights. By doing so, those weights between the target and the irrelevant measurements can be greatly penalized and, thus, lead to an improved tracking accuracy with a low mismatch rate. Moreover, fusing multiple features took advantage of single feature merit and leveraged the corresponding weights.

We experimentally validated our tracker on a variety of scenarios and qualitatively and quantitatively compared our tracker to the conventional GM-PHD tracker, as well as the state-of-the-art trackers. The results demonstrated that our tracker achieved an improvement in precision and accuracy.

However, the processing speed of our tracker was not fast enough, which limited the real-time application. To remedy this, employing a more efficient occlusion handling method will be helpful and will be explored in our future works.
